# High-Responsivity, Low-Leakage Current, Ultra-Fast Terahertz Detectors Based on a GaN High-Electron-Mobility Transistor with Integrated Bowtie Antennas

**DOI:** 10.3390/s22030933

**Published:** 2022-01-25

**Authors:** Zhen Huang, Wei Yan, Zhaofeng Li, Hui Dong, Fuhua Yang, Xiaodong Wang

**Affiliations:** 1 Engineering Research Center for Semiconductor Integrated Technology, Institute of Semiconductors, Chinese Academy of Sciences, Beijing 100083, China; zhenhuang@semi.ac.cn (Z.H.); lizhaofeng@semi.ac.cn (Z.L.); dh0511@semi.ac.cn (H.D.); xdwang@semi.ac.cn (X.W.); 2 Center of Materials Science and Opto-Electronics Engineering, University of Chinese Academy of Sciences, Beijing 100049, China; fhyang@semi.ac.cn; 3 College of Materials Science and Opto-Electronics Technology, University of Chinese Academy of Sciences, Beijing 100049, China; 4 School of Microelectronics, University of Chinese Academy of Sciences, Beijing 100049, China; 5 Department of Electronic Science and Technology, University of Science and Technology of China, Hefei 230026, China; 6 State Key Laboratory for Superlattices and Microstructures, Institute of Semiconductors, Chinese Academy of Sciences, Beijing 100083, China; 7 Beijing Academy of Quantum Information Science, Beijing 100193, China; 8 Beijing Engineering Research Center of Semiconductor Micro-Nano Integrated Technology, Beijing 100083, China

**Keywords:** terahertz detector, leakage currents, plateau-like effect, responsivity, broadband detection

## Abstract

In this study, we fabricated three kinds of terahertz detectors with different leakage currents to analyze the plateau-like effect. The results indicate that the platform becomes increasingly apparent with the decrease in the leakage current. The fabricated device with the lowest leakage current shows a responsivity of 4.9 kV/W and noise equivalent power (NEP) of 72 $\mathrm{pW}/\sqrt{\mathrm{Hz}}$. Further, it can be used for broadband detection between 215 GHz and 232 GHz with a voltage responsivity of more than 3.4 kV/W, and the response time can be up to 8 ns. Overall, the proposed device exhibits high sensitivity, large modulation frequency, and fast response, which indicates its excellent potential for detection and imaging applications in the THz range, including the detection of the 220 GHz atmospheric window.

## 1. Introduction

I recent years, terahertz (THz) technology has been extensively applied in a variety of fields such as military applications, radio communication, climate monitoring, and medical imaging [1,2]. Consequently, the development of an ultra-fast and highly sensitive detector working in the sub-THz and THz regime has garnered significant research attention. Based on the nonlinear properties of two-dimensional electron gas (2DEG), field-effect transistors (FETs) have been verified as effective THz detectors. The FET-based THz (TeraFET) detectors have the advantages of room-temperature operation, high responsivity ($R_{V}$), and low equivalent noise power (NEP) [3]. These detectors have been fabricated with different materials, including Si [4], graphene [5], GaAs [6], and InP [7]. Compared with the above materials, the high-electron mobility transistor (HEMT) based on a GaN heterojunction provides higher 2DEG channel density with unintentional doping, high breakdown voltage, and ultrafast response. All these properties make GaN HEMTs one of the most promising candidates for THz detection.

High responsivity, low-NEP, and high speed are crucial to realize the practical application of THz detectors. By introducing a planar antenna into THz detectors, the THz signals can be efficiently coupled and the sensitivity is improved [8]. Many antennas such as dipole [9], loop [10], and slot-spiral [11] have been utilized. As structures with linear polarization, strong directivity, and high THz-wave coupling efficiency, bowtie antennas have been employed to fabricate high-performance detectors with optical responsivities ($R_{V}$) of 2 V/W [5], 220 V/W [12], and 3.6 kV/W [13]. In addition, the excellent performance of the transistor is the basic premise to ensure the superior characteristics of THz detector, especially for the reduction in the leakage current of the transistor [14].

In this paper, we present an optimized GaN/AlGaN HEMT with a 150-nmong gate and an integrated bowtie antenna as an effective THz detector. Three kinds of devices with different leakage currents ($I_{L}$) were fabricated by different technological processes. The results show that an infinitesimally small $I_{L}$ generates a plateau-like effect in the subthreshold region, resulting in a comparable output response near the threshold voltage ($V_{th}$). The detector with a smaller $I_{L}$ has a stronger photoresponse and a larger peak width. The fabricated device shows a high $R_{V}$ and low NEP. Furthermore, a stable output is maintained at the modulation frequency of 3.7 kHz, and the response time can reach 8 ns. All of these properties indicate that our fabricated device is a potential THz detector for future applications.

## 2. Detection Model and Characterization Setup

**Figure 1:**
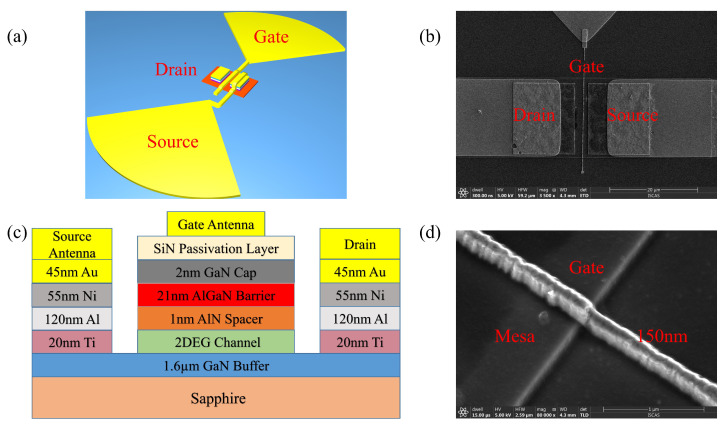
(**a**) Overview of the detection model. (**b**) Local diagram of THz detector at a magnification of 20 μm. (**c**) Specific parameters of HEMT. (**d**) Partial enlarged image of gate across the mesa at a magnification of 1 μm.

The device model used to predict the THz $R_{V}$ and NEP consists of an HEMT and integrated bowtie antenna, as shown in Figure 1a. The fabricated HEMT has epilayers, including a 1.6-μm GaN buffer layer, a 1-nm AlN spacer, a 21-nm AlGaN barrier, and a 2-nm GaN cap layer. It was grown on a 2-inch sapphire substrate. A 2DEG layer was then generated on the top of GaN buffer. The SiN passivation layer was grown in three different ways: sample 1 was prepared by in situ growth through metal–organic chemical vapor deposition (MOCVD), sample 2 was grown by plasma-enhanced chemical vapor deposition (PECVD), and there was no passivation layer on sample 3. The samples grown in the three different ways generated different leakage currents, which were examined as follows.

The device was prepared with the mesa isolation by inductively coupled plasma etching. Source and drain (6 μm × 3 μm) with a gap of 3 μm were aligned, and then e-beam deposition was used to form the metalayers: Ti/Al/Ni/Au (20/120/55/45 nm). This was followed by a rapid thermal annealing at 400/700/870 ${}^{{}^{\circ}}$C (170/40/30 s) to form the source and drain ohmic contact, which provides an unimpeded channel for plasma waves. A bowtie antenna with an optimal radius of 60 μm and radial size of 90 degrees was fabricated by lithography. Finally, a nanogate (150 nm) placed in the center of the gap between source and drain was aligned by electron beam lithography, and then magnetron sputtering was used to form Ni/Au (20/200 nm) metalayers. A schematic of the HEMT is shown in Figure 1c, and the finished device is presented in Figure 1b,d. The two arms of the bowtie antenna were integrated with the gate and source of the HEMT to form an asymmetric structure.

**Figure 2:**
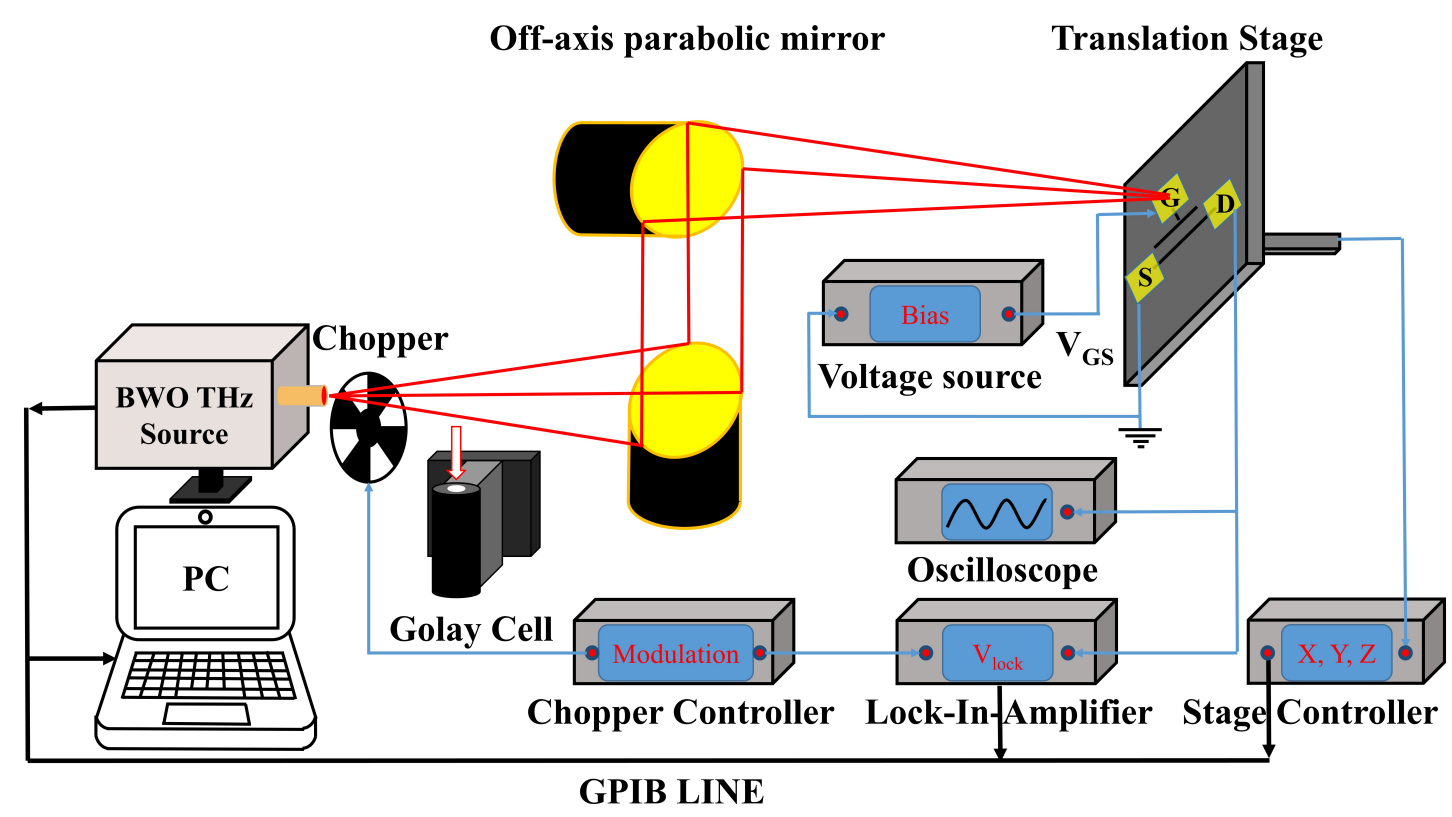
Experimental setup for the characterization of the THz detector.

The experimental setup for the characterization of the detector is shown in Figure 2. Continuous-wave radiation was generated from a backward-wave oscillator (BWO) and focused onto the surface of the detector at the fundamental frequency (175–384 GHz) by two off-axis parabolic (OAP) mirrors (90 degree OAP, Effective Focalength (EFL) = 152.4 mm). The received total power at every electromagnetic frequency was calibrated using a THz Golay cell. The detector was mounted on an FR-4 printed circuit board (PCB) and could be precisely moved by a motorized translation stage. The gate electrode was connected to a DC bias. The source was grounded, and the rectified THz voltage of the detector ($V_{DS}$) was measured using a lock-in amplifier (Stanford Research Systems, SR830). A chopper (SR540, 4–3700 Hz) was used to convert the continuous wave into a square wave, and the modulation signal was simultaneously fed into the lock-in amplifier. A mixed signal oscilloscope (Agilent MSO7104B) was used to record the temporal response before the lock-in amplifier. The data were collected on a personal computer from a General Purpose Interface Bus (GPIB) line. The DC electrical performance of the detector was measured by a semiconductor device analyzer (Agilent B1500A).

## 3. Results and Discussion

The $R_{V}$ and NEP of the THz detectors are defined as follows:

(1) $$\begin{array}{c} \begin{array}{c} R_{V}=\frac{S_{t}\cdot V_{DS}}{S_{eff}\cdot P_{t}} \end{array} \end{array}$$

(2) $$\begin{array}{c} \begin{array}{c} V_{DS}=\frac{\pi }{\sqrt{2}}\cdot \frac{R_{lock}+R_{ch}}{R_{lock}}\cdot V_{lock} \end{array} \end{array}$$

(3) $$\begin{array}{c} \begin{array}{c} \mathrm{NEP}=\frac{N_{V}}{R_{V}}=\frac{\sqrt{4kTR_{ch}}}{R_{V}} \end{array} \end{array}$$

where $S_{eff}$ is the active area of the THz detector, $S_{t}$ is the beam spot size, and $P_{t}$ is the total power of the THz source. The term $\left(R_{lock}+R_{ch}\right)/R_{lock}$ represents the loading effect, which causes the $R_{V}$ drop after the gate voltage ($V_{GS}$) to be less than the $V_{th}$ measured using the lock-in amplifier [15]. $R_{lock}=$ 10 MΩ is the input impedance of the lock-in amplifier, and $V_{lock}$ is the output voltage, which is directly read out from the amplifier. Since the detected signal is a square wave, a factor of $\pi /\sqrt{2}$ must be included in the calculation. In the equation of NEP, $N_{V}$ is the thermal noise of the HEMT, k is the Boltzmann constant, T is the absolute temperature (room temperature = 300 K), and $R_{ch}$ is the channel resistance of the HEMT.

**Figure 3:**
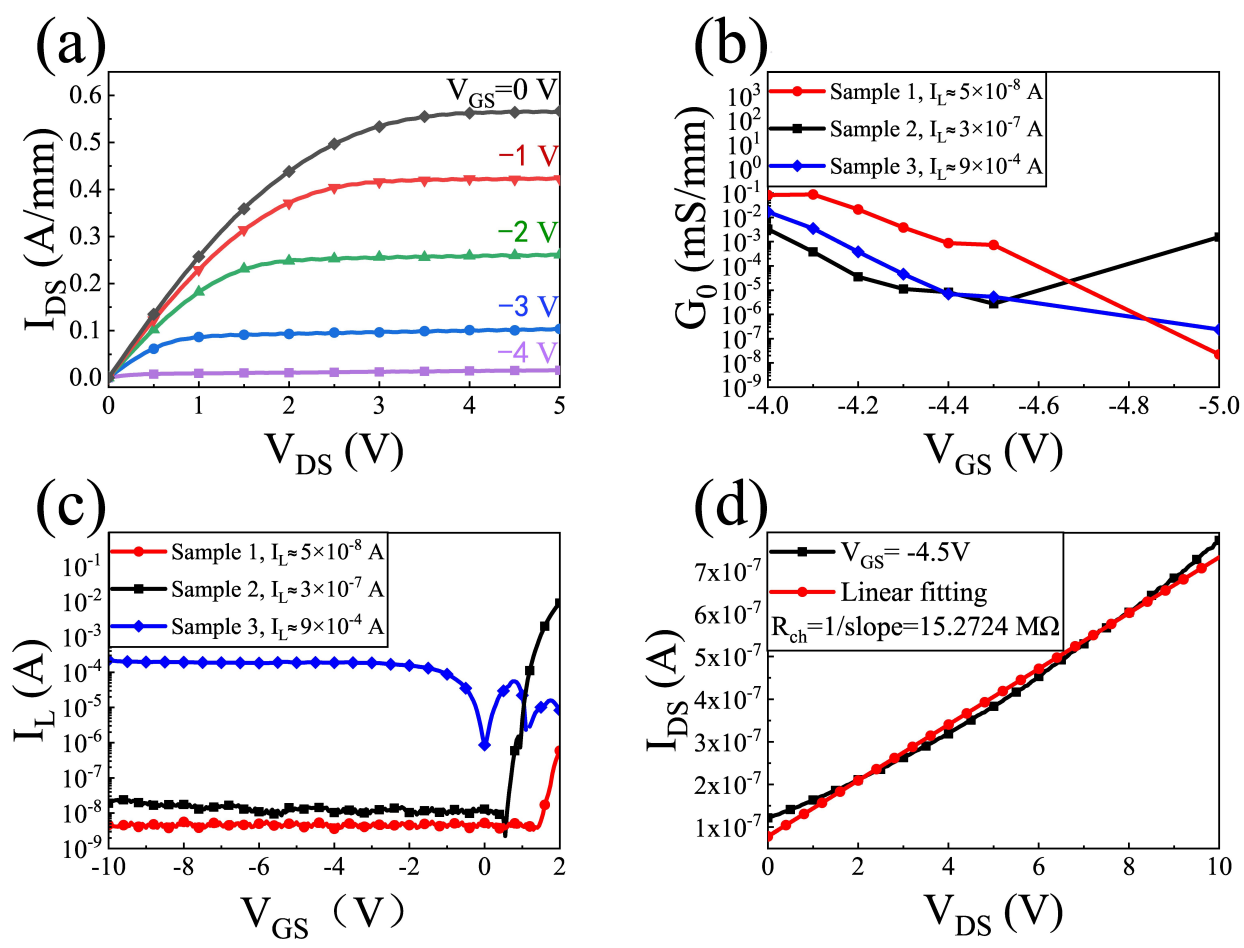
(**a**) I-V characteristics of sample 1. (**b**) Measured source–drain conductance ($G_{0}$) as a function of the $V_{GS}$ in the subthreshold regime. (**c**) Different leakage currents of the three samples. (**d**) Output characteristics of sample 1 at $V_{GS}=-4.5$ V.

Figure 3 shows the DC output, $G_{0}$, and $I_{L}$ characteristics of the GaN/AlGaN HEMT with the integrated bowtie antenna. According to Figure 3a, the $V_{th}$ of the fabricated device is around −4.5 V, and the value of $R_{ch}$ at −4.5 V is 15.2724 MΩ from Figure 3d. The $I_{L}$ corresponding to samples 1, 2, and 3 are 5 × $10^{-8}$ A, 3 × $10^{-7}$ A, and 9 × $10^{-4}$ A, respectively, from Figure 3c.

**Figure 4:**
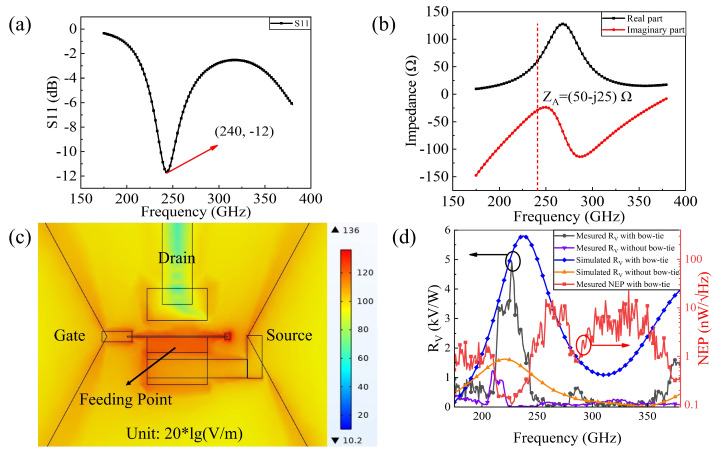
(**a**) The simulated S11 of the structure. (**b**) The simulated impedance of the structure. (**c**) Distribution of the electric field on the surface of the TeraFET detector around the gate (top view). (**d**) Measured and simulated $R_{V}$ with and without a bowtie antenna from 175 to 384 GHz. The red line indicates the measured NEP of the detector with a bowtie antenna. All the results above correspond to sample 1.

By scanning the THz detector through the translation stage, $S_{t}$ was obtained in a range tuned from 19.63 ${\mathrm{mm}}^{2}$ up to 50.27 ${\mathrm{mm}}^{2}$ (the radius of the spot is from 2.5 mm to 4 mm) with the frequency ranging from 175 to 384 GHz. Further, the effective area $S_{eff}$ represented the active antenna dimensions of π × ${\left(60\;\mu m\right)}^{2}$× 0.5. The total power $P_{t}$ was calibrated by a standard Golay cell. All the related parameters were substituted into Equations (1) and (3) to obtain the $R_{V}$ with and without the bowtie antenna from 175 to 384 GHz. The results are shown in Figure 4.

Figure 4a,b indicate that the resonant frequency was 240 GHz, and the impedance was (50-j25) Ω at this point, which met with the design requirement. It is clear from Figure 4d that the maximum $R_{V}$ was 4.9 kV/W at 227 GHz, which is among the best values reported for nonresonant detection by GaN/AlGaN HEMTs integrated with bowtie antennas at room temperature. Furthermore, the simulation results are in excellent agreement with the measured results. The shift in frequency is due to the increased antenna size of the experimentally fabricated device, with the use of a positive photoresist in the lithography process, and the difference in $R_{V}$ may be attributed to the fact that perfect impedance matching cannot be achieved between the antenna and the HEMT in the fabrication process. The $R_{V}$ could be further improved by optimizing the substrate thickness [16]. Further, the minimum NEP was 0.1 nW/$\sqrt{\mathrm{Hz}}$ of $V_{GS}=-4.5$ V. Compared to the device without the bowtie antenna, the $R_{V}$ of the device with integrated bowtie antenna was significantly improved, which is mainly due to the optimized nanogate, proper antenna size, and the asymmetric structure.

The asymmetric structure results in an asymmetrical distribution of the electric field, which leads to a potential difference between the source and drain [13]. In the FET-based terahertz detector system, the whole modulation process takes place in the channel, especially in the region directly regulated by the gate. The extremely short gate can localize the coupled THz energy within a very narrow region, thereby significantly enhancing the modulation of the plasma wave and improving the sensitivity of the detector [8]. This phenomenon is clearly shown in Figure 4c, which was obtained by the commercial software COMSOL; all the related parameters in the simulation were the same as the reality in Figure 1c, and the feeding point of the antenna is shown as an illustration. A lumped port excitation was set between the source and gate electrode, and then the PML (Perfect Matchayer) boundary condition wraps the model inside. In the actual measurement, the characteristics of sample 2 and 3 versus the frequency were in the same variation tendency as sample 1, the differences between them were the maximum responsivity and minimum NEP. Their characteristics versus the $V_{GS}$ were more intuitive, as shown in the following sections.

**Figure 5:**
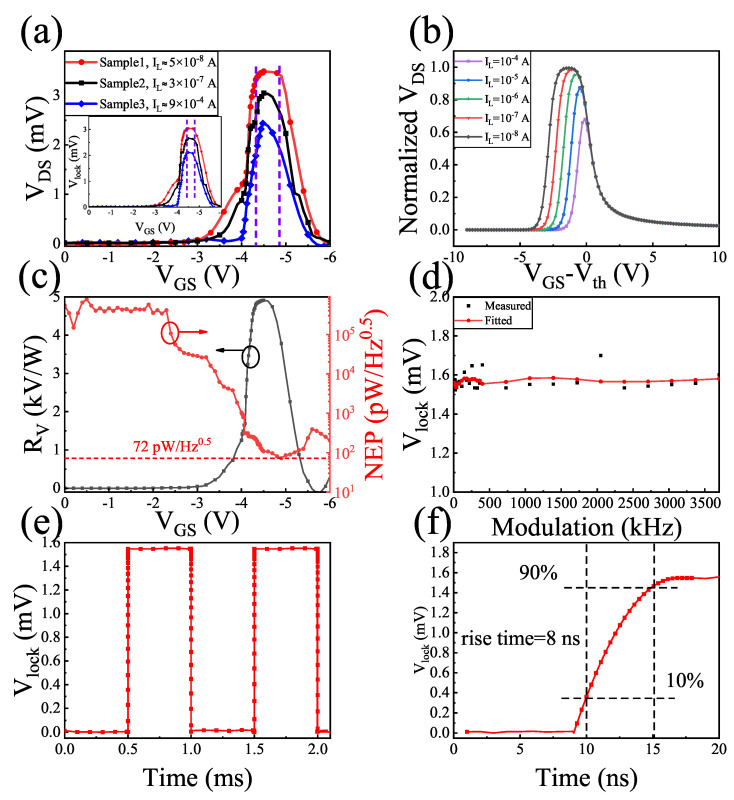
(**a**) Measured $V_{DS}$ versus $V_{GS}$ and $V_{lock}$ curves of the three samples with different $I_{L}$. (**b**) Simulated normalized $V_{DS}$ as a function of the $V_{GS}$ at different leakage currents. (**c**) Responsivity and NEP versus $V_{GS}$ of sample 1. (**d**) Measured and fitted $V_{lock}$ versus amplitude modulation frequency of electromagnetic wave of sample 1. (**e**) Temporal response of the fabricated device chopped by an electronic modulator at 1 kHz of sample 1. (**f**) Measured response time of sample 1.

Figure 5a shows the $V_{DS}$ versus $V_{GS}$ and $V_{lock}$ curves of the three samples with different $I_{L}$. For nonresonant detection, $I_{L}$ is a vital parameter to derive the detector output. $I_{L}$ suppresses the detector response in the subthreshold region, leading to a maximum value in the photoresponse versus the gate voltage. For the same type of HEMTs, the width of the peak can be theoretically calculated [14] as follows:

(4) $$\begin{array}{c} \begin{array}{c} W_{peak}\propto \left(1/\epsilon \right) \end{array} \end{array}$$

where ln(1/ϵ) is a dimensionless parameter, which is a slowly varying function of $I_{L}$. In principle, the smaller the $I_{L}$, the larger the value of ln(1/ϵ), and the larger the peak width. However, from the perspective of the loading effect, this phenomenon occurs because $G_{0}$ (in Figure 3b) kept decreasing when the gate voltage was below the $V_{th}$ in the low $I_{L}$ state; thus, the loading effect became more important according to Equation (2). From the inset picture of Figure 5a, $V_{lock}$ kept a nearly flat state between −4.4 V to −4.6 V even though the $I_{L}$ changed. However, the variation tendency of $G_{0}$ in the subthreshold region varied with the $I_{L}$ (in Figure 3b). For the smallest $I_{L}$ of sample 1, $G_{0}$ would keep decreasing when the gate voltage was below the $V_{th}$; thus, $V_{DS}$ would broaden the peak width between −4.36 V to −4.8 V, as sample 2 of −4.39 V to −4.7 V. The more obvious the reduction trend, the wider the platform. On the contrary, for the larger $I_{L}$ of sample 3, $G_{0}$ increased gradually below the $V_{th}$; in this case, the peak width decreased between −4.39 V to −4.5 V. Furthermore, the measured output increased with the decrease in the leakage current due to the excellent gate-control ability. Further, we also simulated the normalized $V_{DS}$ of different leakage currents, and the result agreed well with the actual situation. The calculation and derivation of the simulation came from the literature [14], and the specific parameters required for the simulation are described in this paper.

The variation trends of $R_{V}$ and NEP as a function of the gate voltage at 227 GHz for sample 1 are presented in Figure 5c. The maximum $R_{V}$ was 4.9 kV/W of −4.5 V, and the minimum NEP was 72 pW/$\sqrt{\mathrm{Hz}}$ of −4.7 V. It can be seen in Figure 5d that the photovoltage remained almost constant in the modulation frequency range of 4–3700 Hz, which was limited by the chopper. The $V_{lock}$ showed noise (at multiples of 50 Hz) due to power line interference. In this case, large noise can be generated when using lock-in technology. The temporal response of the fabricated device was determined when the incident light was chopped at 1 kHz, and the readout voltage was recorded by a mixed signal oscilloscope. The response time of this device is characterized by measuring the time between 10% and 90% of the generated signal, either on the rising or the falling edge [17]. For the measurement of the transient response of the photovoltage, the output port of device was directly connected with the oscilloscope, the lock-in amplifier was not operating at this time. The chopper continued to work, the chopping frequency was 1 kHz, and then the continuous wave converted into the square wave. The oscilloscope displayed the operating status of the device with and without terahertz, and the time of device from on-state to off-state was the transient response time. It is worth noting that the output voltage of the device was around 1.6 mV without the preamplifier, which was larger than the noise generated by devices and the environment. Thus, we could directly read out the output voltage without the lock-in amplifier. There is no denying the fact that the readout voltage response by the oscilloscope was not accurate, but the transient response time was not affected by the noise. The results are shown in Figure 5e,f. The minimum response time of the TeraFET detector was calculated as [18]

(5) $$\begin{array}{c} \begin{array}{c} t_{min}\propto \frac{L^{2}}{\mu V_{GS}}\approx 1.7\times 10^{-10}\;s \end{array} \end{array}$$

Here, *L* is the gate-controlled length (nearly 1 μm from Figure 4a), and μ is the electron mobility (1300 cm${}^{2}$/Vs). The response time of the fabricated device was 8 ns, which is inferior to the theoretical value due to the bandwidth limitation of the oscilloscope.

Under the excitation of the THz electric field, 2DEG with high mobility could complete the signal transmission between source and drain at an extremely high speed. This process was much faster than that in a thermoelectric detector with an electron temperature gradient formed by the electron–electron and electron–phonon interaction [19,20] and in a photodetector based on photo-assisted tunneling [21]. The short response time is an important parameter to define the merit of a THz detector, which is usually desired in high-performance devices. The performance of the fabricated THz detector is comparable or even superior to previously reported detectors (see Table 1).

## 4. Conclusions

In conclusion, we fabricated three kinds of THz detectors with different passivation technologies and analyzed the effect of the leakage current on the device performance. The results revealed the generation of a plateau-like effect, and a higher photoresponse was obtained at a lower leakage current. A high sensitivity of 4.9 kV/W and 72 pW/$\sqrt{\mathrm{Hz}}$ with a low leakage current was achieved by integration with a bowtie antenna, and the response time was up to 8 ns. Furthermore, the device response was superior to that of the existing TeraFET detectors. In addition, the NEP of our fabricated device has improved close to that of the widely-used Schottky diodes detectors [25] and TeraFET detectors and could show excellent characteristics in room temperature. Overall, based on its unique attributes of room-temperature operation, low leakage current, and ultra-fast response, this detector is a promising candidate for future THz applications.

## Figures and Tables

**Table 1:** Comparison of the performance and efficiency of the proposed and previously reported detectors at room temperature.

Architecture	NEP (pW/$\sqrt{\mathrm{Hz}}$)	$R_{V}$ (V/W)	Response Time (ns)	Measurement
This paper	72	4900	8	Lock-in + Oscilloscope
GaN bowtie [13]	40	3600	-	Lock-in
Si bowtie [12]	48	220	-	Preamplifier + Lock-in
GaN bowtie [22]	25	-	-	Lock-in
Graphene spiral [17]	350	28	9000	Preamplifier + Lock-in + Oscilloscope
Si patch [23]	31	-	0.012	Autocorrelation measurement
SiGe patch [24]	-	15	1.7	On-chip amplifier

## Data Availability

The data that support the findings of this study are available from the corresponding author upon request.
